# Myeloid-Derived Suppressor Cells in the Context of Allogeneic Hematopoietic Stem Cell Transplantation

**DOI:** 10.3389/fimmu.2020.00989

**Published:** 2020-05-22

**Authors:** Maud D'Aveni, Anne B. Notarantonio, Allan Bertrand, Laura Boulangé, Cécile Pochon, Marie T. Rubio

**Affiliations:** ^1^Hematology Department, CHRU Nancy, Université de Lorraine, Nancy, France; ^2^Université de Lorraine, UMR 7365 CNRS, IMoPA, Nancy, France

**Keywords:** myeloid—derived suppressor cell, GvH disease, GvT, cellular therapy, allogeneic stem cell transplanation

## Abstract

Myeloid-derived suppressor cells (MDSCs) are innate immune cells that acquire the capacity to suppress adaptive immune responses. In the context of allogeneic hematopoietic stem cell transplantation (allo-HSCT), MDSCs (in the donor graft and in the recipient, after allo-HSCT) might mediate immune suppression through multiple mechanisms. However, it remains unclear how MDSCs can be distinguished from their normal myeloid counterparts in the hematopoietic stem cell donor graft and during immune reconstitution after allo-HSCT in the recipient. Our ability to understand their exact role in allo-HSCT is limited by the absence of a specific gene signature or surface markers for identifying MDSCs among myeloid cells and by their plasticity in different microenvironments. According to various studies, MDSCs might induce transplant tolerance and control graft vs. host disease (GVHD), but their impact on the graft vs. tumor effect (GVT) is not fully understood. In fact, we know that MDSCs commonly expand in patients with cancer, and they are thought to promote hematological malignancy progression. However, little is known about whether depleting them might be an effective strategy for enhancing GVT effects. Here, we review data published over the past 40 years on allo-HSCT to delineate the different MDSC subsets, and their abilities to induce transplant tolerance and preserve the GVT effect. This review will provide a basis for determining whether one MDSC subset might be proposed as the most appropriate candidate for cellular therapies, due to its ability to modulate GVHD.

## Introduction

Allogeneic hematopoietic stem cell transplantation (allo-HSCT) is the only potential curative treatment for some hematopoietic malignant and non-malignant diseases. The success of this therapy is compromised by the development of life-threatening graft-vs.-host disease (GVHD). GVHD is characterized by the activation and proliferation of alloreactive donor T cells that subsequently attack host target organs, such as skin, gut, and liver (1). Immunosuppressive therapies administered to control GVHD are associated with an increased incidence of malignancy recurrence, because they impair the graft-vs.-tumor (GVT) effect. They are also associated with an increased incidence of infections. Therefore, recent strategies are considering whether regulatory cells might be good candidates for minimizing GVHD and maintaining GVT. The main candidates are: mesenchymal stromal cells (MSCs) (2, 3), regulatory T cells (Tregs) (4, 5), invariant Natural Killer T cells (iNKTs) (6), and myeloid-derived suppressor cells (MDSCs) (7).

MDSCs were first described in the peripheral blood, lymphoid organs, spleen, and tumor sites in the settings of cancer, infection, chronic inflammation, and more recently, transplantation and autoimmunity (8). Although MDSCs are barely detectable in the peripheral blood of healthy individuals, the number of circulating MDSCs increases in cancer settings, where they promote immune evasion (9, 10). Currently, MDSCs are attracting interest in the context of human allo-HSCT. These cells can mediate immune suppression through multiple mechanisms: by producing reactive oxygen species (ROS), depleting key amino acids required for T cell proliferation, and producing immunosuppressive cytokines (11). Moreover, interest has increased in identifying MDSCs in hematopoietic stem cell grafts and in the graft-recipient's peripheral blood cells. Unfortunately, in the context of allo-HSCT, MDSC phenotypes are heterogeneous in humans, and inter-study variability is high. Unlike the MDSCs described in the context of cancer (12), in the context of transplantation, we lack a harmonized or standardized definition of MDSCs. Therefore, it has been difficult to identify the best subset of MDSCs for regulating the allogeneic response. Only one study reported that CD1d expression in monocytic MDSCs was a valuable marker for selecting the subset with the highest suppressive activity (13).

This review summarizes the accumulated findings on MDSCs in the context of allo-HSCT. We examine the various MDSC phenotypes across the different studies, their immunosuppressive mechanisms, their advantages and disadvantages in GVHD and GVT, and their potential application to cellular therapy for controlling GVHD.

## First Experimental Evidence of MDSCs and Current Definition

In the late 1970s, a suppressive cell population, called “natural suppressor cells” (NSC), was first identified in human, murine, and rat bone marrow, spleen, and lymphatic tissues. These cells displayed an ability to suppress T-cell responses *in vivo* and *in vitro* without regard to the typical restrictions imposed by the major histocompatibility complex (MHC) (14, 15). NSCs had the morphological features of immature cells in rat bone marrow, and they weakly expressed macrophage and granulocyte antigens. They were rapidly classified as cells of early monocyte lineage, and they were considered a good candidate for modulating GVHD (16).

Oseroff et al. firstly characterized NSCs in newborn and adult mice after total lymphoid irradiation (17). Then, endogenous NSCs were reported to expand in mice after bone marrow transplantation: in an irradiated syngenic mouse model (18), in MHC-matched bone marrow chimeras (19, 20), and in parent-in-F1 bone marrow chimeras (21). These NSCs were lineage negative, that is: they did not express the typical markers for T-cell (Thy1.2 negative), B-cell (surface immunoglobulin negative), or macrophage (Mac-1 and F4/80 negative). Moreover, these NSCs appeared transiently after allo-HSCT (the number peaked in week 3), and they disappeared by week 12 in minor histocompatibility mismatched recipient mice. NSCs were derived from recipient spleens and were considered radioresistant. They inhibited T-lymphocyte proliferation after mitogenic stimulation (19, 20) and after allogeneic stimulation in mixed lymphocyte reaction (MLR) (17, 18, 21). They also protected recipients against GVHD (21).

In the late 1990's, Johnson et al. demonstrated that, early after bone marrow transplantation, spleen cells collected from allogeneic chimeras contained Sca-1^+^ CD11b^+^ cells with immunosuppressive properties, through nitric oxide (NO) production (22). In another context, recipient mice that lacked SH2-containing inositol phosphatase (SHIP^−/−^) displayed a reduced incidence of GVHD after allo-HSCT. This observation was correlated to an elevated number of CD11b^+^ Gr1^+^ cells in the spleen. SHIP is a 5′ inositol phosphatase that hydrolyzes phosphoinositol 3,4,5-trisphosphate, which regulates cell survival in myeloid cells. SHIP^−/−^ mice had 10- to 20-fold higher levels of CD11b^+^ Gr1^+^ cells with immunosuppressive properties compared to wild-type mice (23). Both those studies hypothesized that an immature CD11b^+^ cell subset might explain the *in vitro* and *in vivo* immunosuppressive effects on alloreactive T cells. In the early 2000's, it was noted that NSCs shared many of the characteristics that defined MDSCs in individuals with cancer, including their myeloid origin, their accumulation after irradiation or bone marrow transplantation and their suppressive function. The accumulation of MDSCs in bone marrow transplantation recipients (allogeneic and syngenic) was related to the pro-inflammatory cytokine release that appeared during the first 2 weeks after irradiation. Moreover, this accumulation was related to the later appearance of alloreactive T cells (24, 25). Similarly, MDSCs were observed after donor lymphocyte infusions (DLIs). These MDSCs were further characterized as Ly6G^+^ Ly6C^+^ CD34^−^ Sca-1^−^ CD31^−^ cells, which produced NO in response to interferon-γ (IFN-γ) (26) (Table 1).

**Table 1:** MDSC subsets and their immune suppressive mechanisms observed after conditioning regimen (irradiation) and after HSCT (allogenic or syngenic) in mice.

**References**	**Mouse model (DonorRecipient)**	**NSC or MDSC phenotype**	**Day (D) of first detection**	**T cell proliferation assay**	**Mechanisms**
Oseroff et al. (17)	BALB/C (after TLI)	Non T cell, non B cell, non macrophage (Thy1.2-, 2C2-, Mac1-, F4/80-)	D+5 (after TLI)	**↘MLR**	?
Sykes et al. (18)	B10B10 B10.D2B10.D2 (syngenic)	Non-T cell, non-B cell, non macrophage	Early weeks (after HSCT)	**↘CML**	?
Holda et al. (19)	B10.D2BALB/C B10.D2B10D2F1 (MiHAgs)	Mac1-, Sca-1-, Thy1-	D+7 (after alloHSCT)	**↘mitogenic response** **↘MLR**	?
Maier et al. (20)	B10.D2BALB/C (MiHAgs)	Thy1.2-, IgS- Non adherent to plastic plate	D+10	**↘mitogenic response**	? (inducible mechanism)
Sykes et al. (21)	B10 +/– B10.D2B10 (syngenic +/– mixed with H2 disparity)	Non-T cell, non-B cell, non adherent, asialo GM1-negative syngenic to the recipient	>D+8 After allo and syngenic HSCT)	**↘CML and MLR**	?
Johnson et al. (22)	B10.BRB10.BR (syngenic) B6129F2 or B10.BR AKR (complete H2 disparity)	Thy1.2-, IgS- Mac1 ^low^, Sca-1+	D+10	**↘MLR**	iNOS
Ghansah et al. (23)	C3H AKR (MiHAgs)	CD11b+/Ly6G+/Ly6C+/CD14-/F4/80-/CD11c-	D+21	**?MLR**	NO
Luyckx et al. (24)	B6 B6D2F1 (partial H2 disparity)	Gr-1^+^/CD11b^+^	D+21	**↘MLR**	iNOS?
Wang et al. (25)	B6B6 (syngenic) B6BALB/C (complete H2 disparity)	Gr-1^+^/CD11b^+^	D+14	**↘MLR**	Arg-1 ROS

*Arrows pointing down indicate a reduction in the activity. ?, uncertain; Arg-1, arginase; CML, cell mediated lympholysis; H2, Histocompatibility 2; iNOS, inducible nitric oxide synthase; MDSC, myeloid derived suppressive cells; MiHAgs, Minor Histocompatibility Antigens; MLR, mixed lymphocyte reaction; NO, nitric oxide; ROS, reactive oxygen species*.

***Mouse models and MHC haplotype***:.

*H2^b^: B6 (C57BL/6), B10: C57BL/10SnJ (a substrain of C57BL) and B6129F2*.

*H2^d^: BALB/C, B10.D2: B10.D2/nSn and DBA/2*.

*H2^k^: AKR, B10.BR, and C3H*.

*H2^bd^: B6D2F1 (C57BL/6 × DBA/2) and B10D2F1 (B10.D2 × BALB/C)*.

Currently, these cells are commonly called MDSCs, and they represent a heterogeneous group of cells. Based on the expression of CD49d, two distinct subpopulations have been described in mice: the first was the monocytic CD49d^+^ subpopulation, which also displayed CD11b^+^ Ly6C^+^ Ly6G^low^ CD115^+^ and the second was the granulocytic CD49d^−^ counterpart, which displayed CD11b^+^ Ly6C^−^ Ly6G^high^ CD115^−^. In humans, three distinct MDSC subsets have been described, based on monocytic, granulocytic, and early-stage characteristics. They commonly do not express markers of mature myeloid and lymphoid cells (lineage negative). Thus, monocytic human MDSCs (M-MDSCs) are defined as: CD11b^+^ CD33^+^, HLA-DR^low/−^, and CD14^+^ (27, 28); granulocytic MDSCs (G-MDSCs) are defined as: CD11b^+^ CD33^+^, HLA-DR^low/−^, CD15^+^ but CD14^−^ (29); and early-stage MDSCs (called e- or P-MDSCs) are defined as: CD33^+^, CD11b^low^ HLA-DR^low/−^, CD14^−^ CD15^−^.

## Roles of MDSCs and Mechanisms of Action

### Murine Models of GVHD

**Figure 1:**
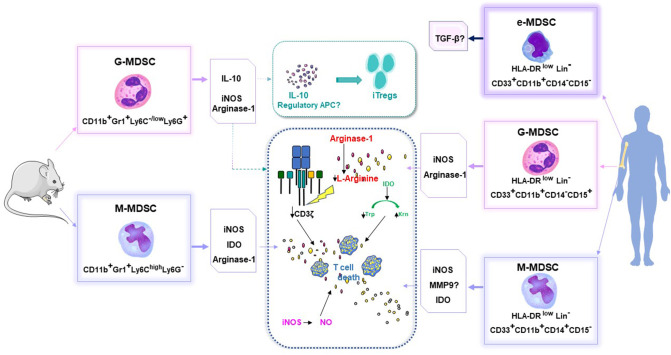
MDSC phenotypes and their capacity to inhibit the proliferation of allogeneic T cells, in mice and humans. Arg-1, arginase; APC, antigen presenting cells; IDO, indoleamine 2,3-dioxygenase; Inos, inducible nitric oxide synthase; iTregs, induced T regulator cells; Krn, kynurenin; Lin, Lineage; MDSC, myeloid derived suppressive cells; M-MDSC, monocytic MDSC; G-MDSC, granulocytic MDSC; E or P-MDSC, early stage MDSC; MMP9, matrix metalloproteinase 9; TGFβ, transforming growth factor beta; Trp, Tryptophan.

In the context of allo-HSCT, MDSCs are mainly defined by their capacity to inhibit the proliferation of allogeneic T cells. To date, these immunosuppressive properties have been attributed to four main mechanisms demonstrated *in vitro* and *in vivo*, including: NO production; arginase 1-mediated L-arginine depletion; indoleamine 2,3-dioxygenase (IDO)-mediated tryptophan (an essential amino acid) conversion; and T regulatory lymphocyte (Treg) induction (Figure 1). Of note, the ROS mechanism of immunosuppression, which was antagonized by adding catalase, was only described once *in vitro* (25).

Experimentally, the immunoregulatory role of NO was established by showing that immunosuppression could be reversed with NO synthase (NOS) inhibitors, such as NG-monomethyl-l-arginine monoacetate, *in vitro*. NO is known to be a negative regulator of intracellular-signaling protein cascades downstream of the interleukin-2 (IL-2) receptor (30, 31). NO blocks the phosphorylation and activation of several signaling proteins, including Janus-activated kinases 1 and 3, signal-transducer and activator of transcription-5, extracellular-signal-regulated kinase, and protein kinase B (32). NO affects the stability of IL-2 mRNA and the release of IL-2 from activated lymphocytes (33). High NO concentrations also induce T-cell apoptosis through various mechanisms, including accumulation of the tumor-suppressor protein, p53; signaling through Fas or tumor necrosis factor (TNF)-receptor family members; or signaling through caspase-independent pathways (34). The main MDSC subset capable of producing NO is the M-MDSC subset (CD11b^+^ Ly6C^high^ Ly6G^low^). We previously demonstrated that, within the M-MDSC population, there was a highly immunosuppressive subpopulation of cells that expressed CD34; these cells were called CD34^+^ monocytes (35). *In vitro*, CD34^+^ monocytes required T-cell-mediated IFN-γ stimulation to produce NO and to inhibit T cell activation and proliferation. *In vivo*, adoptive therapy with CD34^+^ monocytes protected mice from acute GVHD.

Depletion of L-arginine with arginase 1 inhibits T-cell proliferation by inhibiting T-cell expression of the CD3ζ chain, the cell-cycle regulator, cyclin D3, and cyclin-dependent kinase 4 (36). Recently, Highfill and colleagues demonstrated that MDSCs generated from murine bone marrow cells and cultured with granulocyte colony-stimulating factor (G-CSF) and granulocyte-monocyte colony-stimulating factor (GM-CSF) induced arginase-1 activity, which depleted T-cell L-arginine. This depletion resulted in the inhibition of allogeneic T-cell responses, both *in vitro* and *in vivo*. The addition of IL-13 to bone marrow cultures resulted in up-regulating arginase-1 activity, which then increased the suppressive activity of this MDSC subset (37).

IDO is a potent immunoregulatory enzyme that converts the essential amino acid, tryptophan, into catabolic products, collectively known as kynurenines. Joo et al. reported that IFN-γ treatment induced functional IDO activity in MDSCs (Gr1^+^ CD11b^+^), which suppressed allogeneic T-cells, and thus, modulated GVHD (38).

IL-10 is a potent anti-inflammatory cytokine that plays a role in interactions between neutrophils and Tregs (39). Conventional T cells can be converted to Tregs as a consequence of antigen exposure in the periphery, under inflammatory and non-inflammatory (TGF β, IL-10) conditions (40). After allogeneic HSCT, peripheral Tregs emerge *in vivo*, due to IL-10 production by G-MDSCs (41, 42). Induced peripheral Tregs are involved in the control of immunity at sites of inflammation, particularly at mucosal surfaces. More recently, natural Treg proliferation was demonstrated through a mechanism that depended on their cell-cell contact with MDSCs that expressed the Programmed Death-1 ligand (PDL1) (43).

In the context of chronic GVHD, the role of MDSCs has been poorly explored. In a mouse model of chronic GVHD, it was reported that *ex vivo* cultured MDSCs could modulate chronic GVHD presumably by preventing thymic tissue damages and reducing the percentages of CD4+ T cells that produced IL-17 (Th17 cells) and IL-4 (Th2 cells) (44).

### Human allo-HSCT and GVHD (Table 2 + Figure 1)

MDSCs have been observed in grafts of G-CSF-mobilized peripheral blood hematopoietic stem cell (PBSCs) (45). The presence of some MDSC subsets in hematopoietic stem cell grafts has been correlated with a lower incidence of acute GVHD. For instance, common monocytic MDSCs (defined as Lin^−^ HLA-DR^low^/^−^ CD11b^+^ CD33^+^ CD14^+^) (46); CD34^+^ monocytic MDSCs (Lin^−^ HLA-DR^low^/^−^ CD11b^+^ CD33^+^ CD14^+^) (35); and early-stage MDSCs (HLA-DR^−^/^low^ CD33^+^ CD14^−^ CD15^−^) (47) were correlated with a lower incidence of acute GVHD in HLA-matched and haplo-identical HSCT settings (48). Moreover, G-CSF-primed bone marrow grafts contained higher levels of Lin^low/−^ HLA-DR^−^ CD33^+^ CD11b^+^ MDSCs, compared to PBSC grafts, and the G-CSF primed bone marrow grafts were associated with improved GVHD-free survival (49).

**Table 2:** MDSCs in the setting of human allogeneic HSCT.

**References**	**Type of MDSC**		**Early Stage**	**Before/after HSCT**	**Immunosuppressive mechanism**	**Acute GVHD**	**Chronic GVHD**	**OS**	**NRM**	**EFS**
	**Monocytic**	**Granulocytic**								
Luyckx et al. (45)	Lin^−^, HLA-DR^−^ CD11b^+^ CD33^+^	Lin^−^, HLA-DR^−^ CD11b^+^ CD33^+^		Before	?	ND	ND	ND	ND	ND
	CD14^+^ CD15^−^	CD15^+^ CD14^low^								
Vendramin et al. (46)	Lin^−^, HLA-DR^−^ CD11b^+^ CD33^+^			Before	?	↘	ND	ND	ND	ND
	CD14^+^									
D'Aveni et al. (35)	Lin^−^, HLA-DR^−^ CD34^+^ CD11b^+^ CD33^+^ CD14^+^			Before	iNOS	↘	ND	ND	ND	ND
Wang et al. (47)			HLA-DR^−^ CD33^+^ CD16^−^	Before	TGFβ?	↘	↘	=	=	=
Lv et al. (48)	Lin^−^, HLA-DR^−^ CD11b^+^ CD33^+^ CD14^+^		Lin^−^, HLA-DR^−^ CD11b^low^ CD33^+^ CD14^−^ CD15^−^	Before	?	↘	↘	=	=	=
Fan et al. (49)	Total MDSCs:	Lin^−^, HLA-DR^−^,	CD11b^+^ CD33^+^	Before	?	↘	↘	=	=	=
Mougiakakos et al. (50)	HLA-DR^−^ CD14^+^			After	IDO	↗	ND	ND	ND	ND
Guan et al. (51)	CD33^+^ CD15^−^ CD14^+^ HLA-DR^−^	CD33^+^ CD15^+^ CD66b^+^		After	Arg-1 iNOS (for G-MDSCs)	**=**	ND	ND	ND	ND
Kim et al. (52)	CD14^+^ HLA-DR^−^			After	?	↘	ND	ND	**=**	**=** **(↗RI)**
Lee et al. (53)	CD14^+^ HLA-DR^−^			After	MMP9	=	ND	=	↘	↘

*Arrows indicate increases (pointing up) or decreases (pointing down) in the activity or effect (according to the number of MDSC); =, indicates no change observed; ?, unknown; Arg-1, arginase; EFS, event free survival; GVHD, graft vs. host disease; HSCT, hematopoietic stem cell transplantation; iNOS, inducible nitric oxide synthase; MDSC, myeloid derived suppressive cells; MMP, matrix metalloproteinase 9; ND, not done; NRM, non relapse mortality; OS, overall survival*.

After an allo-HSCT, MDSC recovery during immune reconstitution and its clinical relevance have not been fully determined. MDSCs were first observed in recipients that developed acute GVHD. Those MDSCs were defined as a myeloid circulating cell subset (HLA-DR^low/−^ CD14^+^) endowed with *in vitro* suppressive properties through IDO (50, 51). Furthermore, M-MDSCs (HLA-DR ^low/−^ CD33^+^ CD14^+^) and G-MDSCs (HLA-DR^−/low^ CD33^+^ CD15^+^ CD66b^+^) have been isolated from recipients early after transplantation (within 3 months). Those cells could suppress third-party CD4 T-cell proliferation and Th1 differentiation, and they promoted Treg development (51). Increased levels of circulating MDSCs have also been described in patients treated with extracorporeal photopheresis for acute and chronic GVHD (54). Extracorporeal photopheresis was associated with an increase in circulating MDSCs, reduction in CD19^high^ CD20^high^ B cells, and reductions in the expression of CD38 and the BAFF-receptor (55). Unfortunately, the potential mechanisms that gave rise to these observations and the correlations between MDSCs and B cells in the context of chronic GVHD remain unknown.

Prospective studies have attempted to identify which MDSC subsets in recipients could impact post-transplantation outcomes, including overall survival (OS), non-relapse mortality (NRM), event-free survival (EFS), and acute and chronic GVHD. The common findings of those studies were:

- early expansion of G-MDSCs was not correlated with post-transplantation outcomes (52, 53).
- early expansion of M-MDSCs was associated with a higher early infection incidence, a higher NRM, and a lower EFS (and/or higher relapse incidence = RI), but did not affect OS.
- early expansion of M-MDSCs was not associated with acute GVHD in patients that received transplants from siblings or matched unrelated donors (51, 53). Lower expansion of M-MDSCs after allo-HSCT was associated with severe acute GVHD in a cohort of patients that received transplants from siblings, matched unrelated donors, haploidentical-related donors, and double cord donors (52) (Table 2).

To summarize these different studies, M-MDSCs that circulate in the peripheral blood of human recipients early after transplantation can be considered as biomarkers of inflammation, and they can predict some post-transplantation outcomes (infections, GVHD, NRM). However, these findings require validation in larger cohorts.

### Impact of MDSCs on the GVT Effect

In mice, when MDSCs generated *in vitro* were infused to treat severe acute GVHD, the GVT effect was conserved (37). This effect was observed even when the adoptive transfer was performed with MDSCs freshly isolated from tumor-bearing mice. This phenomenon was explained by the observation that MDSCs spare cytotoxic T lymphocytes (NKG2D^+^ CD8 T cells), which play an important role in the retention of GVT activities (56). Moreover, it was demonstrated that CD4 T cells were more susceptible to suppression by MDSCs, and the development of NKG2D^+^ T cell populations was associated with the expression of NKG2D ligands on MDSCs. In another mouse model, MDSCs attenuated GVHD in an IL-10 and Treg-dependent manner; (42) indeed, it was previously shown that Tregs could preserve the GVT effect in mice (57). In some studies, post-transplant chemotherapies [bendamustine (58) and cyclophosphamide (59)] induced CD11b^+^ Gr1^+^ cells which probably played role in transplant tolerance induction. However, the effect of MDSCs on GVT in the model of post-transplant bendamustine was unclear, because chemotherapy can also improve tumor control (58).

In humans, MDSCs have been related to worse outcomes in hematological malignancies (60, 61); therefore, MDSCs were suspected to be involved in relapses after allo-HSCT. However, in the allo-HSCT setting, only a few prospective studies have reported a significantly higher probability of relapse in patients with higher M-MDSC frequencies than other patients, in the 30 days following allo-HSCT (52, 53). Other studies suggested that MDSCs preserved the GVT effect (26, 56). In a recent study, G-MDSCs derived from GCSF-mobilized donors inhibited *in vitro* NK cytolytic activity through an IDO and PGE2 mechanism. Those findings suggested that G-MDSCs had a potent inhibitory effect on the antileukemia activity of donor mature NK cells in recipients (62). For instance, DLIs, which are performed to control hematological malignancies, have been tested with or without G-CSF treatment. The DLIs derived from PBSCs collected after G-CSF treatment favored the conversion to full donor chimerism, despite the higher content of M-MDSCs. Moreover, G-CSF-treated DLIs were associated with lower cumulative incidences of relapse and disease progression, and did not significantly increase the cumulative incidence of GVHD (63). Additionally, during the first 100 days after allo-HSCT, the number of MDSCs in peripheral blood was correlated with the occurrence of severe acute GVHD, but not with an increased risk of malignancy recurrence (64). These observations suggested that MDSCs might not inhibit the GVT effect.

## Are MDSCs of Interest as a Cellular Therapy?

### Generation of MDSCs in Mouse Models (Table 3)

MDSCs can be successfully generated in mice. This was first established with CpG. CpG was administered either alone or emulsified in incomplete Freund's adjuvant. This treatment caused an accumulation of double-positive CD11b^+^ Gr-1^+^ cells in the donor's peripheral blood and spleen. Splenic CD11b^+^ Gr1^+^ cells isolated from treated mice efficiently suppressed alloreactivity in an MLR *in vitro*, and they prevented GVHD *in vivo* (65). Later, Joo et al. administered G-CSF to B6 mice. They observed that the mobilized donor graft spleen used for hematopoietic stem cell transplantation contained a large number of immature Gr-1^+^ CD11b^+^ myeloid cells. These cells suppressed alloreactive donor T cells, which resulted in the inhibition of acute GVHD through an IDO-independent mechanism (38). MacDonald et al. described cells that were induced by progenipoietin-1 (a synthetic G-CSF/Flt-3 ligand molecule). These cells can (retrospectively) be considered MDSCs, because they promoted transplant tolerance by inducing MHC class II-restricted, IL-10-secreting, antigen-specific Tregs (41). In another experimental model, MDSCs were induced after culturing bone marrow cells for 4 days with G-CSF and GM-CSF (37). The resulting CD11b^+^Ly6G^low^Ly6C^+^ MDSCs could inhibit allogeneic T-cell responses *in vitro* and *in vivo* by inducing arginase-1 activity. Addition of exogenous IL-13 in the culture produced a subset of MDSCs (MDSC-IL-13) with greater immunosuppressive potential, due to an up-regulation of arginase-1. MDSC-IL-13 cells migrated to sites of allopriming, where they limited alloreactive donor T-cell proliferation, activation, and pro-inflammatory cytokine production (37). These cells had potent suppressive activity, which resulted in the prevention of acute GVHD lethality. Transplantation of these generated MDSCs inhibited GVHD, but not the anti-tumor cytotoxicity of alloantigen-specific T cells. Moreover, these MDSCs skewed the allogeneic T cell profile toward type-2 T cells, which upregulated T helper 2 (Th2)-specific cytokines (66). Because G-CSF-treated PBSCs are currently used as a source of hematopoietic stem cells in humans, two research teams evaluated the impact of G-CSF-induced MDSCs present in the donor graft. They showed that both G-MDSCs and M-MDSCs induced by G-CSF could reduce acute GVHD in a Treg-dependent manner, through different mechanisms (35, 42) (Table 2).

**Table 3:** MDSCs (cellular therapy) in HSCT mouse models: induction, mechanisms of action, and impact on GVHD, GVL, and OS.

**References**	**MDSC phenotype**	**Factors that induced MDSCs**	**T cell proliferation in MLR**	**Immunosuppressive mechanisms**	**Acute GVHD**	**GVL**	**OS**
Morecki et al. (65)	Gr1^+^ CD11b^+^ CD11c^−^ CD14^−^ F4/80^−^	CpG+IFA	↘	(IL-6, IL-10, IFN-γ)?	↘	ND	↗
Joo et al. (38)	Gr-1^+^ CD11b^+^ Ly6C^+^	G-CSF	↘	IDO	↘	ND	↗
MacDonald et al. (41)	GM cells CD11b^+^ Gr1^low^	G-CSF+FLT3-L		IL-10 ↘Treg	↘	ND	↗
Highfill et al. (37)	CD11b^+^ Ly6G^low^ Ly6C^+^ IL-4Rα^+^ F4/80^+^	G-CSF+GM-CSF +/–IL-13	↘	Arg-1	↘	=	↗
Messman et al. (66)	Gr-1^+^ CD11b^+^ CD115^+^ IL-4Rα^+^	G-CSF+GM-CSF	↘	Th2	↘	=	↗
D'Aveni et al. (35)	CD34^+^ CD11b^+^ CD33^+^ CD115^+^ Ly6C^+^	G-CSF	↘	iNOS Tregs	↘	ND	↗
Perobelli et al. (42)	CD11b^+^ Ly6G^high^ Ly6C^−^	G-CSF	ND	IL-10 Tregs	↘	=	↗

*Arrows indicate increases (pointing up) or decreases (pointing down) in the activity or effect; = indicates no change observed; ?, uncertain; Arg-1, arginase; FLT3-L, fms-like tyrosine kinase 3 ligand; G-CSF, granulocyte colony stimulating factor; GM-CSF, granulocyte monocyte CSF; GVHD, Graft vs. host disease; GVL, Graft vs. leukemia (or lymphoma); HSCT, hematopoietic stem cell transplantation; IDO, indoleamine 2,3-dioxygenase; IFA, incomplete Freund's adjuvant; IL, interleukin; iNOS, inducible nitric oxide synthase; MDSC, Myeloid derived suppressive cells; MLR, Mixed lymphocyte reaction; ND, not done; OS, Overall survival; Th2, Helper T cell type 2*.

### Generation of MDSCs in Humans

Successful treatments with *ex vivo* cultured MDSCs have been reported in mouse models, but what do we know about human GVHD? Generating MDSCs for clinical applications might be difficult, because MDSCs represent a rare subpopulation of myeloid cells. A Korean research team proposed to expand MDSCs from one cord blood unit (CBU). They investigated which cytokine combinations (GM-CSF/SCF) could efficiently expand and differentiate human MDSCs from a culture of CD34^+^ cells. They showed that human MDSCs could be expanded on a large scale, compatible with clinical applications. They produced up to 10^8^ MDSCs (HLA-DR^low^CD11b^+^CD33^+^) from 1 CBU. Infusion of these expanded MDSCs (i.e., third-party cells) significantly reduced GVHD scores, which prolonged survival in a NOD-scid IL2rg^null^ (NSG) xenogeneic mouse model of GVHD (67). Unfortunately, producing sufficient MDSCs required 6 weeks of continuous culture, which might be too long for their use as a treatment for severe acute GVHD. In fact, the most effective approaches for GVHD are those that dampen T-cell responses early after transplantation (prophylaxis). Once alloreactive T cells have begun to contribute to organ injury, it is probably too late to propose cellular therapy. Because most MDSCs do not survive after freeze-thawing, a biobank of these cells is not relevant, and MDSC generation in humans is a difficult avenue of research. To generate a rapidly available cellular therapy, a donor apheresis sample was sorted, and hematopoietic progenitor cells (CD34^+^ cells) were selected and frozen. At the right moment, CD34^+^ cells were thawed and cultured for 20 days with stem cell factor, thrombopoietin, fms-like tyrosine kinase 3 ligand, GM-CSF, and IL-6. This treatment generated both M-MDSCs and G-MDSCs (68). However, these cells were only tested *in vitro*. Alternatively, Bonnotte's team proposed to generate *ex vivo* human suppressor cells of monocytic origin (HuMoSCs), rather than from hematopoietic progenitor cells. These HuMoSCs were obtained after 7 days of culture with GM-CSF and IL-6. Because they were obtained from mature cells, they could not be called MDSCs, but they promoted, *in vivo*, the development of a CD8 T lymphocyte subset that expressed FOXP3, which induced peripheral tolerance. Interestingly, these cells could be infused to prevent GVHD on day 7 after transplantation. Alternatively, they could be frozen, then thawed at the precise moment they were needed (69). Unfortunately, little is known about the *in vivo* viability, trafficking, and expansion of these cultured cells.

## Discussion and Future Considerations

MDSCs represent a diverse population of immature myeloid cells that are currently well-described in the context of allogeneic stem cell transplantation. MDSC properties have been described according to observations at different times during the transplantation procedure: in the donor graft after mobilization, in the recipient after a conditioning regimen, and during the reconstitution of the donor-derived immune system. Flow cytometry studies have analyzed MDSCs in the graft and in recipient blood samples during post-transplantation immune recovery; however, their role is not fully understood. They represent a biomarker of inflammation, but their immunosuppressive properties are not always maintained; thus, these properties are likely to depend on the context.

MDSCs were first described as highly immunosuppressive cells derived from radioresistant recipient cells. However, recent data have suggested that the intestinal microbiota might provide new avenues of researches on how MDSCs might be induced and modulated after a conditioning regimen. We know that conditioning regimens induce tissue damage, which allows bacterial products to translocate from the skin and mucosa into the blood, where they can activate immune cells. After conditioning, lipopolysaccharide (LPS) mediates activation of the pathway involving Toll Like Receptor 4 (TLR4)-myeloid differentiation primary response gene 88 (MyD88)- nuclear factor-kappa B (NF-κB). In a recent study, recipients that received MyD88-deficient bone marrow cell transplantations developed severe intestinal GVHD associated with an insufficient expansion of donor MDSCs. That result suggested that intestinal microbiota might regulate MDSC induction through the TLR/MyD88 pathway (70). In humans, patients that received broad spectrum antibiotics (carbapenem) developed dysbiosis of the intestinal microbiota. This condition was associated with reduced recovery of E- and M-MDSCs and an increase in the cumulative incidence of intestinal GVHD (71). Those findings suggested that, early after conditioning, the TLR/Myd88 pathway might first stimulate (signal 1) Nlrp3 inflammasome, which might induce the generation of MDSCs.

On the other hand, the conditioning regimen also provides a second stimulus (signal 2), through damaged tissues, which cause the release of damage-associated molecular pattern (DAMP) molecules. The release of DAMPs in the conditioning phase can activate the Nlrp3 inflammasome in recipient cells, and this activation correlated to an increased risk of severe GVHD (72). The main endogenous DAMP molecule, Adenosine-5'-triphosphate acts as a ligand for the P2X7 receptor (73). Activation of the P2X7 receptor was shown to be a critical step in the pathogenesis of GVHD (74). Koehn et al. recently demonstrated that the binding of ATP to the P2X7 receptor could induce the assembly and activation of the Nlrp3 inflammasome. Full activation of the Nlrp3 inflammasome led to MDSCs differentiation and the loss of suppressor function (75). Thus, MDSCs that are induced early after conditioning could rapidly differentiate and lose their immunosuppressive function after allogeneic HSCT. Indeed, pharmacologic agents that targeted Nlrp33 and the P2X7 receptor provided significantly higher survival, due to a lower incidence of GVHD (7, 76).

Strikingly, excessive production of mitochondrial ROS molecules was observed during conditioning; this represents another second stimulus (signal 2) of the Nlrp3 inflammasome (77). In hematology, as in oncology, the metabolism of MDSCs in the tumor microenvironment is commonly described as highly active. These MDSCs produce high levels of cytosolic ROS, due to the action of NADPH oxidase (NOX2). This environment appears to maintain MDSCs in an undifferentiated state with immunosuppressive properties (78). In allogeneic HSCT, NOX2-mediated ROS production in MDSCs has been poorly described (25). However, excessive oxidative stress, due to both mitochondrial and cytosolic ROS production, could compromise redox homeostasis in MDSCs. This condition can activate a nuclear factor called erythroid-derived 2-like 2 (Nrf2), which regulates an endogenous antioxidant mechanism that is involved in the regulation of various pathways in MDSCs, such as metabolic reprogramming and differentiation (79). Therefore, further studies on MDSCs are warranted in the context of allo-HSCT that aim to decipher the roles and interactions of specific metabolism/redox signaling molecules and inflammasome. Those findings could improve our understanding of the fine-tuning and maintenance of the immunosuppressive functions and differentiation of MDSCs.

To summarize, the generation (67–69) and depletion of MDSCs are gaining interest in the field of research that aims to shape the balance between GVHD and GVT. Therefore, MDSC metabolism and the inflammasome pathway should be studied carefully. Results from those studies might determine whether we should consider the administration of repeated infusions of *ex vivo* expanded MDSCs to modulate GVHD, as is currently routinely performed in cellular therapy with MSC (80).

## Conclusion

The present review showed that MDSCs are gaining interest in the context of allo-HSCT. They represent a key regulatory cell type in the inflammatory environment induced by allo-HSCT, particularly in the context of GVHD. Although murine studies have revealed a plethora of mechanisms and pathways that give rise to the generation of MDSCs and their suppressive functions, evidence of these putative mechanisms in humans remains scarce. In the context of allogeneic HSCT, MDSCs have mainly been described as inhibiting the activation, proliferation, and function of T cells. However, few studies have demonstrated MDSCs interacting with other cells, such as NK cells, iNKT cells, Dendritic Cells, or B cells. Based on this review, we would like to highlight that, to date, M-MDSCs seem to be the most well-described, well-understood MDSC subset in the context of allo-HSCT. The M-MDSC subset is highly conserved between species (mouse and human), has powerful immunosuppressive properties, and could be produced in *ex vivo* cultures. Future studies on MDSC metabolism and the inflammasome pathway might pave the way for new pharmacologic strategies that can either dampen or enhance MDSC suppressor function according to the clinical context.

## Author Contributions

MD'A and AN drafted the manuscript. CP, AB, LB, and MR reviewed and edited the manuscript.

## Conflict of Interest

The authors declare that the research was conducted in the absence of any commercial or financial relationships that could be construed as a potential conflict of interest.
